# Gender-Based Disparities in Nursing Compensation

**DOI:** 10.1097/AJN.0000000000000347

**Published:** 2026-07-23

**Authors:** Jessica Leiberg

**Affiliations:** **Jessica Leiberg** is the dean of the School of Nursing and an associate professor at Concordia University Wisconsin in Mequon, WI. Contact author: jessica.leiberg@cuw.edu. The author has disclosed no potential conflicts of interest, financial or otherwise.

**Keywords:** compensation disparities, gender pay gap, policy reform, workforce equity

## Abstract

This article explores the *Wisconsin 2024 RN Workforce Survey Report*'s findings, which suggest persistent, systemic pay disparities among women, men, and nonbinary nurses in the state. It highlights compensation disparities in the Wisconsin RN workforce due to gender and identifies patterns across health care settings, specialties, and roles that can be used to inform strategies promoting pay equity. Addressing this inequity will require enforcement of current laws, transparency in wages and compensation structures, equity-focused organizational policies, and continued research using micro and longitudinal data.

**Figure:**
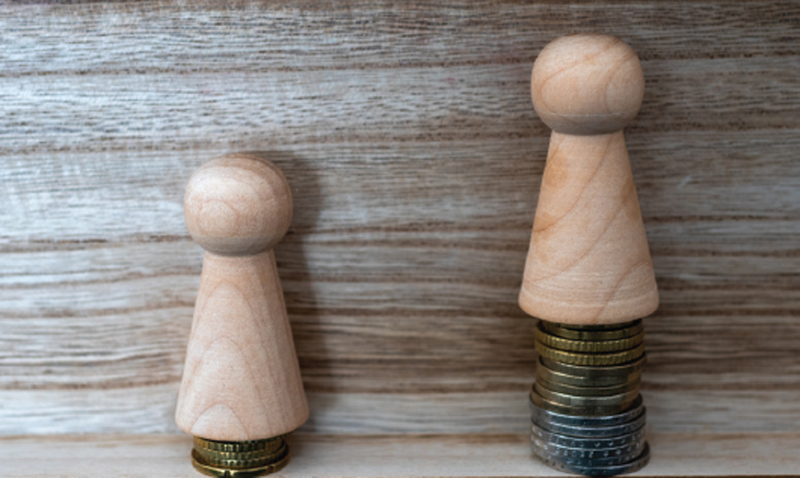
Photo by piu_IX / Shutterstock.com.

Despite the nursing profession's foundational commitment to equity, compassion, and holistic care, disparities in compensation persist.^1^ These inequities are particularly difficult to address given the demographic composition of the nursing workforce. Nationally, the RN workforce is predominantly made up of women—an estimated 89.3%—with male RNs comprising 10.4% and nonbinary RNs 0.4% of the workforce.^2^ The *Wisconsin 2024 RN Workforce Survey Report* reveals similar findings: approximately 91.5% of those surveyed report being women, compared with 8.2% identifying as men and 0.2% as nonbinary or other.^3^ This gender imbalance, while reflective of nursing's historical roots as a female-dominated profession, paradoxically coexists with a gender pay gap that the Wisconsin workforce survey's findings reveal to favor men across nearly all settings and specialties.^3^ This article explores the published findings of the *Wisconsin 2024 RN Workforce Survey Report*,^3^ disaggregated by gender, workplace, specialty, and role, with the aim of illuminating patterns of inequality to potentially inform policy and organizational strategies promoting compensation equity.

## GENDERED-PAY INEQUITY

The persistence of gendered-pay inequity in health care is not new. As health care providers professionalized in the 20th century, they inherited and institutionalized broader societal norms that undervalued women's labor despite legislation designed to promote equal pay.^4^

Even as educational requirements, clinical responsibilities, and leadership opportunities have expanded, compensation structures have not kept pace with the profession's evolving complexity, nor have they adequately addressed gender-based pay disparities.^1^ In Wisconsin, for instance, men in nursing, though a numerical minority, are more likely to occupy higher-paying roles, ascend to leadership positions more rapidly, and negotiate higher starting salaries, according to the findings of the workforce survey.^3^ Research across professional fields has documented that gender pay gaps often persist, not through direct-pay discrimination alone but also through the disproportionate representation of women in lower-graded roles within the same occupation.^5^ In nursing, this dynamic takes a distinctive form, in which men have historically benefited from the “glass escalator” effect, advancing more rapidly into higher-compensated leadership positions.

Growing recognition of nonbinary and gender-diverse people in the nursing workforce raises additional concerns about inequity. Emerging evidence from the broader labor market suggests that nonbinary individuals are more likely to experience earning disparities and limited access to advancement opportunities.^6^ There is reason to believe similar patterns may extend to nursing, as workforce surveys have started to capture labor market information about this population.^2^,^3^ These disparities are compounded by additional demographic factors, such as race, ethnicity, and age, which further stratify income and career entry and mobility, or lack thereof, within the profession.^7^,^8^

Addressing pay inequities requires more than awareness; it demands structural change.^9^ Transparent salary aggregates, equity audits, mentorship programs for underrepresented groups, and inclusive leadership development initiatives are essential steps toward a more just and equitable workforce.^10^

## EXPLORING THE FINDINGS OF THE REPORT

I explored the authors' findings in the *Wisconsin 2024 RN Workforce Survey Report* regarding responses to several questions, including Question 8 (“Please indicate any of the clinical areas listed below in which you have specialized knowledge and/or experience of two or more years”), Question 14 (”What was the primary [most frequent] setting in which you provided care to people infected with Covid-19?”), Question 45 (“Please estimate your 2023 pre-tax annual earnings for your primary place of work. Include overtime and bonuses but exclude sign-on bonuses”), Question 48 (“Which one of the following best describes your functional or employment position role at your primary job?”), and Question 75 (“What is your gender?”).^3^ The survey findings categorized median annual pretax earnings by gender (woman, man, nonbinary), primary place of work, nursing specialty, and functional role and analyzed racial and ethnic identity and age. For this article, I analyzed the *Wisconsin 2024 RN Workforce Survey Report*'s descriptive statistics, which are included in that report in table format, looking for common themes and stand-out findings.^3^ Many interesting themes regarding pay disparities emerged, particularly in terms of gender, workplace setting, and specialty care.

## GENDER-BASED DISPARITIES

The findings of the *Wisconsin 2024 RN Workforce Survey Report* reveal that men consistently earn more than other nurses, nonbinary nurses consistently report the lowest earnings, and the pay gap widens with advanced education.

**Men report higher median earnings**. The results of this survey reveal a consistent and pervasive trend: men in nursing earn more than women and nonbinary nurses across nearly every workplace setting and clinical specialty.^3^ This pattern holds true in hospitals, ambulatory care, correctional facilities, and even in traditionally lower-paying environments, such as community care. For example, according to the survey results, in ambulatory care settings men reported median annual earnings in the $85,001 to $95,000 range, whereas women and nonbinary nurses said they earned $65,001 to $75,000—a gap of up to $30,000 annually.^3^

*Hospitals and correctional care*. Primary places of work located in hospitals and correctional care settings yielded the highest median earnings, particularly among survey respondents who identify as men. These settings also frequently offer pay differentials due to elevated safety risks, underserved and hard-to-recruit locations, higher patient acuity, increased knowledge of specialized care, 24/7 staffing requirements, higher turnover, and overtime needs. Another potential factor could be the increased leadership roles within hospital systems. (The *Wisconsin 2024 RN Workforce Survey Report* contains information about gross income and doesn't stratify base and additional pay.^3^)

*Specialty care*. Differences in pay among people who identify as men, women, and nonbinary vary by specialty area. The results of the Wisconsin survey show that nurses working in anesthesia and family health have among the highest earnings. Nurses identifying as men who worked in anesthesia care reported earning $155,000, whereas women reported earning $95,001 to $105,000, a potential salary difference of more than $50,000 per year.^3^ Family care nurses reported diverse incomes, with those identifying as men saying they earned $105,001 to $115,000, whereas women said they made $85,001 to $95,000 and nonbinary nurses reported earning the least in this specialty, from $75,001 to $85,000.^3^

Notably, among nurses working on medical–surgical, intensive care, and psychiatric units, men reported being paid a full income bracket more than women and nonbinary individuals. Among all specialties, labor and delivery stands out as the most egregious example of gender-based income disparity. In this specialty, men reported median earnings of $155,000—more than double the $75,001 to $85,000 range reported by women, according to the survey results.^3^ This is particularly striking given that labor and delivery is a field overwhelmingly staffed by women and associated with women's health.

**Nonbinary nurses consistently earn less**. Nonbinary nurses reported the lowest earnings across nearly all specialties, with some of the starkest figures emerging in addiction and substance abuse care, where median annual salaries in Wisconsin were reported to be $30,000, compared to $85,001 to $95,000 for those identifying as men or women.^3^ This is significantly below the median pretax annual earnings for RNs nationally in 2024, which was $88,000,^2^ suggesting a pattern of economic marginalization.

**Pay gap widens as education increases**. Advanced practice nursing requires a master's degree in nursing compared to a bachelor's or associate degree for an RN. An increase in pay is generally associated with an advanced education; however, the pay gap also widens for nurses who have advanced degrees in Wisconsin.^3^ According to the Wisconsin survey results, the median annual earning range for nurses who identify as women within advanced practice roles was $115,001 to $125,000, whereas for men it was $155,000.^3^ Of note, $155,000 was the highest median annual earning amount listed in this survey, so men reporting this income category may have been earning more.

This finding is also seen among nurses with doctoral level degrees. Within this category, the survey results show that nurses who identify as men reported that their median annual earnings ranged from $135,000 to $145,000, whereas women reported earning $105,001 to $115,000 and nonbinary people said their median annual income was $85,001 to $95,000.^3^

## DISCUSSION

Gender-based wage gaps persist, even in female-dominated professions, suggesting systemic biases in role assignment, negotiation, and promotion.^5^ The findings of the *Wisconsin 2024 RN Workforce Survey Report* reveal a pattern that is suggestive of gender-based, structural compensation differences among nurses in the state.^3^ Additionally, the underrepresentation and lower earnings of nonbinary nurses point to broader issues of inclusion and equity.^1^

Disparities in pay are not merely a reflection of role or experience. The findings of widening gaps in pay among those with advanced education suggest that men in nursing are more likely to be fast-tracked into higher-paying specialties and leadership roles. Men also benefit from societal perceptions that associate male presence with authority and competence in clinical settings, which can influence hiring, promotion, scope of practice, and salary negotiations.^11^ These systemic advantages contribute to the wage gap evidenced in the *Wisconsin 2024 RN Workforce Survey Report*, which persisted even when controlled for education and years of experience.^3^

Several factors contribute to these disparities. Men may be more likely to experience differential treatment and different practice allowances toward shift assignments, overtime opportunities, skills, or access to high-paying opportunities.^12^,^13^ The magnitude of this gap raises critical questions about equity in role distribution and compensation practices within gendered specialties.

The underrepresentation of nonbinary people in nursing data makes it difficult to draw broad conclusions, but the available evidence suggests systemic exclusion.^3^,^14^ Nonbinary nurses may face barriers similar to those documented among gender-diverse workers in other professional settings, including discrimination in hiring, limited mentorship, and reduced advancement opportunities.^15^ Additionally, a lack of inclusive workplace policies and support systems can create environments in which nonbinary professionals are undervalued or pushed into lower-paying roles.^15^

Federal protections prohibit gender-based pay inequity. The Equal Pay Act of 1963 and Title VII of the Civil Rights Act of 1964 require equal compensation for substantially equal work and prohibit sex-based wage discrimination.^16^,^17^ Subsequent legislation, including the Lilly Ledbetter Fair Pay Act of 2009, has attempted to strengthen enforcement by extending the timeframe for filing claims.^18^ State-level statutes further reinforce these protections, yet gender pay disparity persists. Additionally, health care systems can be penalized if they receive federal payment from Medicare or Medicaid and are found guilty of gender pay inequity. Additionally, employers who violate equal pay laws may be required to provide back pay, liquidated damages, and compensatory relief, in addition to paying attorney's fees and making court-ordered policy changes.^16^,^17^,^19^

The nursing pay inequity revealed in the *Wisconsin 2024 RN Workforce Survey Report* underscores the importance of intersectional analysis. Gender identity, in combination with other factors such as race, age, and role, can compound disadvantages and limit economic mobility. Addressing these disparities requires a call to action including intentional efforts to create inclusive policies, the collection of disaggregated data, and assurance of equitable access to high-paying roles for nurses of all gender identities.^15^ Last, existing equity laws require more consistent and rigorous enforcement.

## IMPLICATIONS

The findings of the *Wisconsin 2024 RN Workforce Study* are troubling, and the longitudinal implications are great. For instance, a male nurse who earns $25,000 a year more than a nurse who identifies as a woman or nonbinary person will make $125,000 more over the course of five years, not accounting for the potential increased opportunity for promotion. Over the course of a career, a male nurse could earn $1,000,000 more than a woman or nonbinary nurse (again, not accounting for potential promotion opportunities). These differences can affect overall quality of life, future educational opportunities, a nurse's ability to provide for children or a family, the ability to retire, and much more. This example using $25,000 is also a modest comparison point, particularly considering that pay differences reached more than $70,000 per the findings of the *Wisconsin 2024 RN Workforce Study*.^3^ These findings could, hopefully, be a catalyst for an increase in annual pay for women and nonbinary nurses that is equal to that of male nurses.

## FOR FUTURE STUDY

Access to the *Wisconsin 2024 RN Workforce Study*'s microdata for deeper analysis would allow for a more targeted evaluation and recommendations. The lack of longitudinal data in this survey doesn't allow for the analysis of whether the pay gap is worsening or improving. Allowing for additional evaluation of the success of current and past efforts and policies—or lack thereof—would be worth evaluation. Promoting advancement and salary equity within nursing could potentially decrease levels of burnout and turnover, as well as the number of nurses intending to leave the profession.^20^

Addressing wage disparities in nursing requires targeted interventions, including transparent pay structures, bias training, enforcement of already existing laws, and support for underrepresented groups in leadership and high-paying specialties. Future research should use an intersectional approach to determine what has shaped, is shaping, and will shape nursing career trajectories and compensation.
